# Informed consent and coercion in recruitment advertisements for oocyte donors

**DOI:** 10.1186/s12905-024-03302-w

**Published:** 2024-09-20

**Authors:** Ruby Lake, Isa Berzansky, Andrea Lanes, Serene Srouji, Elizabeth Ginsburg, Iris Insogna

**Affiliations:** 1 University of California Los Angeles, Los Angeles, CA USA; 2 Department of Obstetrics and Gynecology, Brigham and Women’s Hospital, 75 Francis Street, Boston, 02115 MA USA; 3 Columbia University Fertility Center, New York, NY USA

**Keywords:** Egg donation, IVF, Oocyte donors, Infertility, Informed consent

## Abstract

**Background:**

As the use of donor eggs for in vitro treatment has increased, both medically affiliated and private donor egg agencies have turned to online advertisements to recruit donors. The American Society for Reproductive Medicine provides recommendations encouraging ethical recruitment of donors, however there is no formal regulation for the informed consent process for egg donor recruitment and compensation. Underrepresentation of risks and targeted financial incentives may pose a risk to the informed consent process.

**Methods:**

Data from online advertisements for egg donors active between January 1 - August 31, 2020, were collected to analyze content related to risks, Covid-19 precautions, donor payment, and desired donor characteristics. Advertisements for egg donors on Google, Craigslist, and social media were analyzed. Primary outcomes included the mention of the risks of egg donation, including the risk of Covid-19 exposure, in donor egg advertisements. Secondary outcomes included language targeting specific donor characteristics and financial compensation.

**Results:**

103 advertisements were included. 35.9% (37/103) of advertisements mentioned some risk of the egg donation process, and 18.5% (19/103) mentioned risks or precautions related to Covid-19 exposure. Of advertisements for private donor egg agencies, 40.7% (24/59) mentioned any risk, compared to 29.6% (13/44) of medically affiliated egg donation programs; the difference was not statistically significant (p-value = 0.24). Agencies targeting students and donors of a specific race were more likely to offer payments over $10,000 for an egg donation cycle. Among advertisements offering over $20,000 for donor compensation, 72.7% (8/11) recruited women under the age of 21.

**Conclusion:**

Egg donor recruitment advertisements, for both medically affiliated programs and private agencies, were unlikely to mention risks including the risk of exposure to Covid-19. Non-medically affiliated private donor egg agencies were more likely to violate multiple American Society for Reproductive Medicine ethics guidelines, including offering higher than average compensation, and recruiting donors from young and vulnerable populations.

## Background

The rising utilization of donor eggs for in vitro fertilization (IVF) has expanded options for family building, bringing with it both medical and societal benefits, as well as a unique set of ethical considerations. [1] IVF is an assisted reproductive technology that involves fertilizing an egg with a sperm to create an embryo. Egg donation involves using an egg from another individual, rather than one’s own egg, in addition to sperm from a partner or donor, to create an embryo. Many people may require egg donation for family building including women with failed autologous IVF cycles, diminished ovarian reserve, iatrogenic infertility after treatment for cancer, peri or postmenopausal women, men in same sex couples among others. Egg donors may be non-identified (anonymous) or directed (known) donors. The latter group may include friends or appropriate family members of the recipient. The regulation of egg donation varies widely on an international scale, with some countries having minimal governmental restrictions, others mandating anonymous or altruistic donation only, and some countries that have banned it completely. [2] In the United States, the limited regulation and commercialization of egg donation creates several central ethical concerns including the targeted recruitment of financially vulnerable students and other young women, the prioritization of donors with specific characteristics, and the failure to include risk language in early recruitment stages.

Currently, private donor egg agencies in the United States supplement the work of IVF clinics by recruiting egg donors for an ‘agency fee’ charged to the intended parents who contract with them. Private donor egg agencies generally have no medical professionals on staff and connect the donors they recruit with intended parents and external medical facilities. Both medically affiliated donor egg programs and non-medically affiliated private donor egg ‘agencies’ have extended their reach through online advertising. Thus, examining online egg donation advertisements provides an important opportunity for exploring how these entities uphold, or in some cases disregard, ethical standards and expectations.

The process of egg donation is physically intensive and requires a commitment of time. Egg donors undergo extensive medical screening to determine their eligibility. This includes answering an in depth questionnaire regarding personal medical, psychological and family history, blood work and a physical examination involving a breast and pelvic exam. Egg donors undergo approximately two weeks of stimulation with injectable gonadotropins and an ultrasound-guided egg retrieval under anesthesia, in which a needle enters the ovary through the vaginal wall to aspirate ovarian follicles and collect eggs. [3] Though incidence of serious complications is generally low, they include ovarian hyperstimulation (OHSS), ovarian torsion, hemorrhage, infection, intra-abdominal injury, and exposure to anesthesia. [4,5] Potential psychological risks associated with egg donation include breach of anonymity, concerns over future disclosure to offspring, psychological or physical side effects related to temporary hormonal changes, and regret. Prior survey data suggest egg donors do not have an adequate understanding of potential long-term risks of egg donation. [6] With the advent of direct-to-consumer DNA testing, facial recognition software and the expansion of artificial intelligence, donor anonymity can no longer be guaranteed. The ability of donor-conceived persons to identify a donor in the future is quite possible, though many donors may not understand or anticipate this. Within the context of the Covid-19 pandemic, additional risks include increased exposure to the virus. Egg donation requires travel to clinics (often involving air travel) as well as frequent entry into medical facilities for appointments. Underrepresentation of risks within advertisements poses a threat to the informed consent process and autonomy of potential egg donors.

No laws regulate the informed consent process for egg donation recruitment and compensation in the United States. Donor agencies and clinics largely self-regulate, guided in part by the recommendations of the American Society for Reproductive Medicine (ASRM). ASRM guidelines suggest a minimum age of 21years for egg donors and advise that advertisements include a discussion of risks. [7] Disproportionate financial targeting of specific vulnerable populations raises concerns about the commodification of reproduction, the unequal distribution of financial pressures to donate, and the notion that certain individuals’ time and health are worth more than others. Currently, donor recruitment agencies regularly offer upwards of $60,000 for high-demand donors with qualities such as strong test scores, enrollment at Ivy League universities (a small group of elite academic institutions in the United States), or racial or ethnic backgrounds. [8] Despite ASRM guidelines, a 2013 study found that over half of surveyed newspaper and Craigslist advertisements listed minimum ages below 21 years. [9] In 2014, a review of 435 donor recruitment advertisements found low rates of risk disclosure for clinics throughout the US. [10] Similarly, it has been demonstrated that programs often provide inaccurate or incomplete risk information in preliminary donor phone call inquiries. [11] One study found that only one-third of potential egg donors knew about the possibility of ovarian hyperstimulation, only one-fifth knew about the potential for unknown long-term risks, and only one in eight knew about the potential risks of anesthesia exposure with egg retrieval. [12] The process of informing donors on these risks begins with language in early advertisement and recruitment and continues through to donation.

During the pandemic, which had devastated the financial and medical stability of millions, careful consideration of informed consent and financial coercion were of heightened concern. A renewed investigation of egg donor recruitment advertisements was necessary to assess the inclusion of language that targeted students and other vulnerable sub-populations, as well as the omission of language that addressed potential risks of egg donation, particularly within the context of Covid-19. [8] The objective of this study is to compare the mention of risks, including exposure to Covid-19, in egg donor recruitment advertisements from medically affiliated donor egg programs and non-medical private egg donor agencies. Secondarily, it examines how advertisements vary in financially incentivizing donors with desired characteristics. This information may help to inform future guidelines or regulation to reduce financially exploitative advertising and preserve the autonomy of egg donors.

## Methods

This study aims to test the hypothesis that, compared to medically affiliated donor egg programs, private donor egg agencies are less likely to mention risks, including exposure to Covid-19, in their advertisements, and the hypothesis that online advertisements offer greater financial incentive for donors with specific physical or intellectual characteristics. Advertisements for egg donors were reviewed and coded for content of information and language. This study included egg donation advertisements posted on Facebook and Instagram (“social media”), Craigslist, and Google from July 1, 2020 to August 31, 2020. These posts were required to be active within the study inclusion timeframe or embedded within a website that had been updated within the year 2020. Egg donation programs and agencies were only included if they were primarily based within the United States and targeted donors residing within the United States. Agencies were included if they had international branches to their donation program, although no advertisements posted specifically to recruit donors outside of the United States were used for data collection.

Advertisements were classified as Craigslist advertisements, social media advertisements, or Donor Information Pages linked through Google or social media (“Donor pages”). Using “searchcraiglist.org”, key terms within an online library that contains all active Craigslist advertisements across the United States were queried. Social media advertisements were selected using the Facebook “Ad Library”, which enables key terms within all active Facebook and Instagram advertisements to be searched. These two media platforms are part of the same company; therefore, their advertisements were combined, and advertisements were selected if they were available on both platforms. [13] Search terms used included “egg donor”, “egg donation”, “oocyte donor”, and “oocyte donation”. Data were coded separately for information included directly within social media ads (social media) as opposed to information included in ‘donor recruitment pages’ which were embedded in company websites and linked through social media or by google search result links classified as Advertisements. Data were only coded for the information found on the first page which appeared, excluding any information from other sections of the company’s website or social media page. If there was a drop-down menu or an option to scroll through a rotation of images within the same advertisement page, this was noted and recorded. Duplicated advertisements on the same platform were excluded, while unique advertisements posted by the same company on different platforms were included once per platform.

Data collection from the advertisements included a range of financial compensation offered for donors, targeted donor characteristics (age, racial/ethnic group, weight, student achievements, physical traits, mental health history, etc.); mention of counseling services; mention of bonus incentives (free travel, free healthcare testing, and cash bonuses for referring friends); mention of physical risks; mention of COVID-19 related safety precautions; emotional appeals to the altruism; and provision of information regarding the procedure itself. The risk categories were grouped into heightened risk of unplanned pregnancy for donors taking fertility hormones (pregnancy risk), physical risks associated with the egg retrieval process (procedural risk), psychological risks associated with donation (psychological risk), the risk of ovarian hyperstimulation (OHSS risk), and the inclusion of a statement about the potential for unknown/unstudied long-term risks (long-term risk). Indirect targeting of students included demonstrating a preference for students, including highlighting the ability to work with student schedules or claiming to be a flexible second job for working students. Direct targeting was defined as directly requesting students with specific characteristics or describing egg donation as an effective way to repay student debt.

Advertisements were collected from both ‘medically affiliated’ donor egg programs, defined as those associated with a specific IVF clinic or medical facility, as well as from ‘private donor egg agencies’ functioning independently from any medical facility. The staff pages for each agency and clinic were studied to ensure that all programs classified as medically affiliated had reproductive endocrinology and fertility specialists on staff and were able to complete the IVF cycle and egg retrieval in their facility. Frequencies and proportions were calculated for all categorical variables and chi squared tests were performed to determine statistical significance. Fisher’s exact tests were performed when there were small cell sizes. Statistical significance was determined at an alpha = 0.05. All statistics were performed using SAS version 9.4 This study was submitted to Partners Institutional Review Board, and it was determined that approval was not required since this study did not collect human subject data and assessed only publicly available websites

## Results

150 advertisements were identified, and 103 advertisements met inclusion criteria for the study, with 15.5% (16/103) posted on social media (Facebook/Instagram), 38.8% (40/103) on Craigslist, and 45.6% (47/103) on a Donor Page (accessed by clicking a link on Google or social media).

Table 1 depicts the proportion of advertisements posted on social media, Craigslist, or a Donor Page that mentioned any risks, specific risks broken down by type, as well as the availability of psychological and legal counseling for donors. Of all the advertisements included, the majority did not mention any type of risk associated with egg donation. Donor pages embedded in websites contained information on at least one risk of egg donation more often as compared to social media or Craigslist advertisements. A similar pattern was found for advertisements that described the egg donation procedure. The offer of legal counseling or health counselling was infrequently mentioned in social media or Craigslist advertisements, but was stated on 44.7% (21/47) of Donor pages. Of all advertisements, risks associated with hormone treatment, risk of pregnancy, and long-term risks were the most likely to be mentioned while OHSS and procedural risks were rarely mentioned. Additionally, only 1 advertisement directly mentioned any psychological risk. Appeals to altruism were included in 95.2% (98/103) of all surveyed ads.

**Table 1:** Risk and Counseling Information Across Advertisement Type

	All Ads *N* = 103 *n* (%)	Social Media *N* = 16 *n* (%)	Craigslist *N* = 40 *n* (%)	Donor Page *N* = 47 *n* (%)	*p*-value
**No mention of any risk**	66 (64.1)	15 (93.8)	34 (85.0)	17 (36.2)	< 0.01
**No mention of long-term risks** ^**1**^	86 (83.5)	16 (100.0)	39 (97.5)	31 (66.0)	< 0.01
**No mention of OHSS risk**	89 (86.4)	16 (100.0)	40 (100.0)	33 (70.2)	< 0.01
**No mention of pregnancy risk**	85 (82.5)	16 (100.0)	39 (97.5)	30 (63.8)	< 0.01
**No mention of procedural risk**	90 (87.38)	16 (100.0)	39 (97.5)	35 (74.5)	< 0.01
**No mention of hormone associated risk**	85 (82.5)	16 (100.0)	39 (97.5)	30 (63.8)	< 0.01
**No mention of legal counseling**	81 (78.6)	16 (100.0)	39 (97.5)	26 (55.3)	< 0.01
**No mention of health counseling**	69 (67.0)	16 (100.0)	34 (85.0)	19 (40.4)	< 0.01
**No description of procedure**	71 (68.9)	15 (93.8)	38 (95.0)	18 (38.3)	< 0.01

^1^Long-term risks include the advertisement stating the potential for unknown and under-studied long-term risks of taking hormones to stimulate egg production. If an advertisement mentioned the phrase ‘unknown risk’ or ‘long-term risk’ it was included

Table 2 compares medically affiliated donor egg programs to private donor egg agencies without any medical affiliation. No significant relationship was demonstrated between donation program type and the mention of any risk information (*p* = 0.24). Medically affiliated program advertisements were less likely to directly or indirectly target students as compared to private agency advertisements (*p* < 0.01). Private agencies were more likely than medically affiliated programs to request donors of a specific racial or ethnic background, in addition to more often requested specific non-medical physical features (eye color, model characteristics, etc.). Private agencies were also more often willing to pay over $10,000. Of all surveyed advertisements, 81.6% (84/103) contained no mention of Covid-19 despite travel risks associated with the pandemic. Private agencies were also significantly more likely to promote travel as an added benefit or even a central reason to donate (i.e., ‘Take your donation vacation to the sunny west coast!’) (*p* = 0.01).

**Table 2:** Advertisement Content Across Donation Program Type

	All donor egg programs/agencies *N* = 103 *n* (%)	Donor Branch at Medically Affiliated Program *N* = 44 *n* (%)	Non-Medically Affiliated Private Donor Agency *N* = 59 *n* (%)	*p*-value
**Any Risk Mentioned**		13 (29.6)	24 (40.7)	0.24
**Covid-19 Advisory**:				
*No Information*	84 (81.6)	30 (68.2)	54 (91.5)	< 0.01
*Pop-up Banner*	17 (16.5)	13 (29.6)	4 (6.8)	
*Information within Ad*	2 (1.9)	1 (2.3)	1 (1.7)	
**Requests Specific Appearance**	16 (15.5)	1 (2.3)	15 (25.4)	< 0.01
**Requests Specific Ethnicity/Race**	37 (35.9)	8 (18.2)	29 (49.2)	0.02
**Student Target**				
*Directly Targets Students*	18 (17.5)	4 (9.1)	14 (23.7)	< 0.01
*Indirect Targets Students*	36 (35.0)	9 (20.5)	27 (45.8)	
**Targets High Achieving Students**	19 (18.5)	2 (4.6)	17 (28.8)	< 0.01
**Travel Bonus Mention**	50 (48.5)	15 (34.1)	35 (59.3)	0.01
**Donor Payment stated**				
*< $10*,*000*	47 (45.6)	29 (65.9)	18 (30.5)	< 0.01
*> $10*,*000* *< $20*,*000*	37 (35.9)	9 (20.5)	28 (47.5)	
*> $20*,*000*	11 (10.7)	3 (6.8)	8 (13.6)	
*Compensation not specified*	8 (7.8)	3 (6.8)	5 (8.5)	
**Donor Age** ^1^				
*Only recruits 21+*		16 (43.2)	15 (28.8)	0.16
*Recruits under 21 (18*,* 19*,* or 20+)*		21 (56.8)	37 (71.1)	
*Age requirement not provided*		7	7	

^1^Statistical significance only tested among groups with an age stated within advertisement

Table 3 compares the financial compensation offered for specific donor characteristics. Ninety-five advertisements were included; 50.5% (48/95) offered >$10,000 per donation cycle. 19.1% (9/47) of advertisements offering a maximum payment of $10,000 highlighted financial incentives for specific racial and ethnic groups, while 54.2% of advertisements offering greater than $10,000 highlighted a priority for specific racial or ethnic groups. The targeted age groups and different level of compensation offered were not statistically significant (*p* = 0.09). 48.9% (23/47) of advertisements offering less than $10,000 recruited donors under the age of 21; 54.1% (20/37) that offered between $10,000 and $20,000 recruited women under the age of 21, and 72.7% (8/11) that offered over $20,000 targeted women as young as 18 years of age.

**Table 3:** Compensation offered in relation to desired donor characteristics

	Total *N* = 95^1^ *n* (%)	≤ $10,000 offered *N* = 47 *n* (%)	>$10,000, <$20,000 *N* = 37 *n* (%)	>$20,000 *N* = 11 *n* (%)	*p*-value
Prioritized Race/Ethnicity					
**None Specified**	66 (69.5)	38 (80.9)	17 (45.9)	5 (45.5)	< 0.01
**Asian**	19 (20.0)	2 (4.3)	10 (27.0)	5 (45.5)	
**Asian and Caucasian**	3 (3.2)	1 (2.1)	2 (5.4)	0 (0)	
**Asian and Jewish**	2 (2.1)	0 (0)	2 (5.4)	0 (0)	
**Caucasian**	4 (4.2)	3 (6.4)	1 (2.7)	0 (0)	
**Jewish**	5 (5.3)	0 (0)	4 (10.8)	1 (9.1)	
**“Culturally diverse” but unspecified**	4 (4.2)	3 (6.4)	1 (2.7)	0 (0)	
**Student Target (%)**					
**Yes**,** Indirectly**	34 (35.8)	15 (31.9)	17 (46.0)	2 (18.2)	0.03
**Yes**,** Directly**	17 (17.9)	6 (12.8)	10 (27.0)	1 (9.1)	

^1^Advertisements with no information or unclear information on donor payments (*n* = 8) were excluded from the analysis.

## Discussion

The results do not support our primary hypothesis as advertisements by both medically affiliated donor egg programs and private donor egg agencies were unlikely to include information regarding the general risks associated with egg donation. Most advertisements failed to mention potential risks associated with donation, psychological wellbeing, or Covid-19 in any level of detail in early recruitment stages. Our secondary hypothesis was supported by our data, as private donor egg agencies were less observant of age guidelines and compensation recommendations shared by the ASRM and more likely to offer greater financial compensation for donors with specific characteristics.

The underrepresentation of risk found in this study is consistent with previous studies examining donor recruitment, which found risk communication to be inaccurate or incomplete. Additionally, a qualitative review of egg donation advertisements revealed that emotional appeals to potential donors can frame information in ways which may bias them into overlooking risks and challenges. [14] The ASRM has explicitly advised that donors under age 21 not be recruited to avoid exploitation of vulnerable young women, [3] however more than half of all advertisements surveyed here recruited donors under 21. These results are consistent with previous findings from Alberta et al. [9] who found over half of newspaper and Craigslist advertisements listed minimum ages below 21. In the early 2000s, the ASRM suggested that egg donor compensation not exceed $5,000 ($10,000 in exceptional cases) to cover the time, discomfort, and risks of donation. [8] However, the 2011 Kamakahi v. ASRM lawsuit resulted in a court ruling that formal price caps on donor compensation constitute an unlawful restraint of trade, in violation of US antitrust laws. [8] This ruling has been controversial, as it allows agencies and clinics to offer compensation rates that can be considered exploitative when targeting younger age groups, especially those entering an unstable job market during a student debt crisis. Non-medical private agencies often coupled requests for younger donors and donors with specific traits with significantly higher financial incentives, regularly offering over $10,000 and even over $20,000 per cycle.

There is growing literature regarding the needs of donor-conceived persons, emphasizing the importance of disclosing the circumstances of their conception early on in childhood [15,16]. When this information is concealed, and later uncovered, it can be psychologically traumatizing. As donor-conceived persons learn about their conception, some may desire to uncover their donor to learn more about their genetic link, and potentially discover donor siblings. [17] Even if a donor intends to remain anonymous, increasing access to technologies such as individual at-home genetic testing and facial recognition software may make that impossible. Donors may underestimate the psychological and legal consequences of agreeing to forgo parental rights and future contact with children born to intended parents. Additionally, it is impossible to know what donor conceived children may want to ask their biological parent, but that it conceivably include what the donor was paid to donate. Donor anonymity can no longer be guaranteed. These considerations need to be carefully reviewed with egg donors to ensure they understand the possible long term repercussions, and to set appropriate expectations for the future.

The combined effects of unrestricted financial rewards, targeting students, and poor risk communication may create an environment of undue inducement or coercion influencing a person’s autonomous choice. [9] All advertisements were collected during months where Covid-19 was well documented as affecting travel and work behavior throughout the country [18] and the failure to incorporate adequate safety disclaimers while advertising free travel demonstrates a lack of consideration for the unique ethical concerns of offering travel-based employment during a pandemic.

Agency advertisements demonstrate a lack of regard for the blurred ethical boundaries surrounding assigning more monetary value to the eggs of specific sub-categories of women, suggesting that certain women’s time, effort, and willingness to risk their physical safety is worth more than that of others. Given widespread financial difficulties being faced across the country during this pandemic, donor recruiters should be more mindful than ever of the risk of coercive and biased enrollment when offering disproportionately high pay to young students and other potentially vulnerable subpopulations to repay debt. [19] Additionally, there are fertility clinics that are providing patients with the option to perform a “freeze and share” cycle, where they are permitted to maintain ownership of a portion of their frozen eggs in place of financial compensation. [20,21] Such incentive based programs disproportionately target financially disadvantaged patients who do not have the means to afford egg freezing independently.

Limitations of this study include its short duration, the potential influence of the COVID pandemic on advertisement design at the time of the study, and the fact that only the initial page of the advertisement was investigated. This study characterizes only the primary language used to appeal to donors and does not reflect information the programs and agencies may or may not provide within later donor appointments. However, the information prioritized within this initial donor recruitment stage is often the determining factor in potential donors’ assessments of whether to engage with applications, and the rest of the donation process can also be indicative of a company or clinic’s overarching values. Further research should investigate whether disregard for psychological risks persists throughout the donation process.

## Conclusions

Donating one’s eggs to help others build a family is a uniquely generous act that deserves appropriate recognition, and significant yet regulated compensation. Questions are raised regarding the regulation of egg donation, especially as non-medically affiliated private donor agencies are increasingly utilized. If introduced to risk information later in the process, donors may be more likely to minimize or overlook potential risks, than if this information was provided at the outset. When advertisements contain little to no risk language, young women may become heavily invested in the financial and emotional benefits of donation, in addition to added benefits such as travel, free fertility treatments, and genetic testing. Many advertisements contain direct links to donor applications, through which donors can apply into a program before having seen any detail of the donation procedure and the physical and emotional commitments. In accordance with the sunk cost fallacy, individuals are often more likely to continue an endeavor they would not otherwise commit to in order to avoid wasting previously invested resources. To avoid infringing upon donor autonomy, medical clinics and donor agencies can not overlook the possibility of violating informed consent in all phases of donor recruitment: advertisements, interviews, counseling, and assessment. For both medical and non-medical donation programs, this could involve strictly enforcing age guidelines for donation, shifting away from significant disparities in payment for donors, and incorporating accessible risk language.

## Data Availability

The datasets used and/or analyzed during the current study are available from the corresponding author on reasonable request.

## Abbreviations

IVF: In vitro fertilization
OHSS: Ovarian hyperstimulation
ASRM: American Society for Reproductive Medicine
